# Prediction and Optimization of the Long-Term Fatigue Life of a Composite Hydrogen Storage Vessel Under Random Vibration

**DOI:** 10.3390/ma18030712

**Published:** 2025-02-06

**Authors:** Xiaoshuang Xiong, Wentao Wang, Xiang Li, Fei Fan, Jiacheng Zhou, Mingzhang Chen

**Affiliations:** 1 Hubei Key Laboratory of Digital Textile Equipment, Wuhan Textile University, Wuhan 430200, China; xsxiong@wtu.edu.cn (X.X.); ffan@wtu.edu.cn (F.F.); 2 School of Mechanical Engineering & Automation, Wuhan Textile University, Wuhan 430200, China; 2215373006@wtu.edu.cn; 3 China Special Equipment Inspection and Research Institute, Beijing 100029, China; 4 Hubei Key Laboratory of Advanced Technology for Automotive Components, Wuhan University of Technology, Wuhan 430070, China; chenmingzhang@whut.edu.cn

**Keywords:** composite hydrogen storage vessel (CHSV), random vibration, free and constrained modes, fatigue life, numerical simulation

## Abstract

A composite hydrogen storage vessel (CHSV) is one key component of the hydrogen fuel cell vehicle, which always suffers random vibration during transportation, resulting in fatigue failure and a reduction in service life. In this paper, firstly, the free and constrained modes of CHSV are experimentally studied and numerically simulated. Subsequently, the random vibration simulation of CHSV is carried out to predict the stress distribution, while Steinberg’s method and Dirlik’s method are used to predict the fatigue life of CHSV based on the results of stress distribution. In the end, the optimization of ply parameters of the composite winding layer was conducted to improve the stress distribution and fatigue life of CHSV. The results show that the vibration pattern and frequency of the free and constrained modes of CHSV obtained from the experiment tests and the numerical predictions show a good agreement. The maximum difference in the value of the vibration frequency of the free and constrained modes of CHSV from the FEA and experiment tests are, respectively, 8.9% and 8.0%, verifying the accuracy of the finite element model of CHSV. There is no obvious difference between the fatigue life of the winding layer and the inner liner calculated by Steinberg’s method and Dirlik’s method, indicating the accuracy of FEA of fatigue life in the software Fe-safe. Without the optimization, the maximum stresses of the winding layer and the inner liner are found to be near the head section by 469.4 MPa and 173.0 MPa, respectively, and the numbers of life cycles of the winding layer and the inner liner obtained based on the Dirlik’s method are around 1.66 × 10^6^ and 3.06 × 10^6^, respectively. Through the optimization of ply parameters of the composite winding layer, the maximum stresses of the winding layer and the inner liner are reduced by 66% and 85%, respectively, while the numbers of life cycles of the winding layer and the inner liner both are increased to 1 × 10^7^ (high cycle fatigue life standard). The results of the study provide theoretical guidance for the design and optimization of CHSV under random vibration.

## 1. Introduction

Hydrogen energy is extensively considered the most promising candidate to replace fossil fuels in the field of transportation, with the advantages of zero greenhouse gas emissions, no pollution, and renewability, and it is especially widely used in long-distance and heavy-duty trucks [1]. CHSV is one key component of the hydrogen fuel cell vehicle, which is directly related to the driving range, production cost, and safety of the vehicle [2]. During the actual vehicle driving cycle, a lot of uncertainty, such as braking and turbulence, occur randomly, which will cause stress concentration and excessive deformation at the connection of each component of the CHSV, resulting in vibration fatigue failure and a reduction in service life [3]. Therefore, it is of great importance to carry out the random vibration damage analysis, fatigue life prediction, and structure optimization of CHSV for the long-term operation of hydrogen fuel cell vehicles.

In the past few decades, much research has been conducted on the structural design and optimization of CHSV to improve safety performance. Bouhala et al. summarized relevant simulation models and proposed innovative methods for designing CHSV with high performance [4]. A finite element model was developed by Moskvichev et al. [5] to simulate the stress-strain behavior of CHSV with different numbers of composite layers under internal pressure. Their work investigated the effect of the propagation of initial defects in composite shells and the critical size of surface cracks. Kabiri et al. [6] implemented the Tsai-Hill failure criterion into the finite element model of CHSV to study the stress distribution and the critical region and then investigated its fatigue life using the residual strain energy method. Vafaeesefat et al. [7] proposed a multi-objective strategy for the head shape and winding angle optimization of CHSV, taking into account the internal pressure and volume, the vessel weight, and the composite material properties. Furthermore, Wang et al. [8] further proposed a prediction method for the CFRP CHSV under cyclic fatigue loading and high-temperature conditions based on the integration of micromechanics of failure and the time-temperature superposition principle method. Roh et al. [9] established a finite element model of CHSV to analyze the stress distribution among the vessels with a new integrated end-cap design for the dome section. They found that the high-stress points shifted from the dome to the cylindrical section of the vessel with the integrated end-cap design. In addition, Cho et al. [10] further investigated the effect of dome curvature on the failure mode of CHSV through hydraulic burst tests and numerical simulations. To study the complex phenomenon of plastic liner blistering during the depressurization of type IV hydrogen storage tanks, Yersak et al. [11] developed a polymer liner blistering model, which greatly reduced the experimental cost. Halm et al. [12] developed a finite element model to simulate the coupled effects of mechanical damage and temperature on the burst and leak of type IV hydrogen storage tanks under fire tests, which can accurately predict the time to burst of the composite tank, as well as the transition between burst and leak. Rohit et al. [13] conducted a comprehensive investigation into the structural and explicit performance of three types of CHSVs using 3D models for drop and crash tests. The study revealed that Type 1 CHSVs failed to meet safety requirements under extreme conditions, while Type 3 CHSVs exhibited induced stresses below the yield point in both scenarios, and Type 4 CHSVs presented the best performance. Li et al. [14] investigated the hydrogen fill rate, ambient temperature, volume, and hydrogen inlet temperature to evaluate their effects on the temperature rise within the CHSV. Zhou et al. [15] conducted impact damage and bursting experiments on Type III and Type IV cylinders and found that with the increase in impact energy, the fiber fracture area, matrix crack area, and impact depth of the two cylinder types increased. Furthermore, in the same impact energy, Type III cylinders presented a smaller impact depth but a larger fiber fracture and matrix crack area. Hu et al. [16] conducted finite element modeling of CHSV based on the Hashin damage model and metal plastic damage theory [17,18] and carried out blasting tests. Their studies showed that the progressive damage model can effectively investigate the matrix cracking and fiber fracture and predict burst pressure.

Although the finite element modeling and experiment tests have been applied successfully to the structural design and optimization of CHSV, most of them are focused on the performance analysis and service life prediction of CHSV under the inter pressure condition, the leaking condition, and the fire condition. Nevertheless, the CHSV often inevitably suffers vibrations from uneven road surfaces and internal vibrations during vehicle operation. Therefore, it is imperative and of great importance to study the dynamic response and fatigue life analysis of CHSV under random vibration. Yao et al. [19] proposed an approach to optimize the strapping parameters for in-vehicle CHSV using a GA-XGBoost model, and finite element models were established to verify the optimized results. Huang et al. [20] used the finite element method to conduct the multi-objective optimization of stiffness and strength of a two bottle horizontal hydrogen supply system under vibration conditions. Coskun et al. [21] conducted a study on CHSV with various geodesic dome profiles, analyzing modal and random vibration responses. They examined five polar opening radii to reveal the effects of filament winding orientation and internal pressure severity on the dynamic characteristics. Ryu et al. [22] analyzed the effect of the hydrogen shaking phenomenon inside the CHSV by performing the frequency characteristics of the CHSV at zero frequency, intrinsic frequency, and train operation. As the frequency increased, the free surface wave of liquid hydrogen became shorter and more pronounced. Wang et al. [23] further investigated the intrinsic frequency and mode shapes of CHSV, adopted four calculation methods to estimate the vibration fatigue life of the CHSV inner liner, and found that the number of cycle times of the CHSV inner liner calculated by the four methods all reached over 10^6^ cycles. It is not difficult to see from the above research that the structural optimization of the hydrogen supply system and the fatigue analysis of the inner liner of CHSV under random vibration have been widely discussed, but few studies have been conducted on the fatigue analysis and structural optimization of the composite winding layer of CHSV under random vibration.

In this paper, finite element models were developed to simulate the dynamic response of CHSV under random vibration. Modal testing experiments were conducted to verify the accuracy of numerical simulations. Then, the stress and deformation simulation for CHSV under random vibration was performed for the fatigue life prediction, including the stress and deformation analysis of the aluminum liner and composite winding layer. The Steinberg’s method and the Dirlik’s method were used to predict the fatigue life of CHSV under random vibration. In the end, the optimization of ply parameters of the composite winding layer was conducted to improve the stress distribution and fatigue life of CHSV. The results of the study provide a simulation strategy and an experimental method for the prediction and optimization of fatigue life of CHSV under random vibration.

## 2. Methods

### 2.1. Basic Theory of Stochastic Processes with Power Spectral Density (PSD)

Stochastic processes can be classified into two categories according to their statistical properties over time: smooth random vibrations and non-smooth random vibrations. The power spectral density (PSD) can be used to both describe the smooth process in the frequency domain and express the energy distribution of the stochastic process, which is an important parameter of the stochastic process in the frequency domain [24] and is defined as follows:

(1) $$S_{x}(\omega )=\int_{-\infty }^{+\infty }R_{x}(\tau )e^{-j\omega \tau }d\tau \;$$

(2) $$R_{x}(\tau )=\frac{1}{2\pi }\int_{-\infty }^{+\infty }S_{x}(\omega )e^{j\omega \tau }d\omega$$

where $S_{x}(\omega )\;$ is the self-power spectral density function, ω and *f* are the circular frequency and frequency, respectively, and $R_{x}(\tau )$ is the autocorrelation function.

In the engineering application, the circular frequency ω>0; therefore, the unilateral power spectral density is defined as follows:

(3) $$G(\omega )=\{\begin{array}{cc} 2S_{x}(\omega ), & \;\omega >0\\ S_{x}(0), & \;\omega =0 \end{array}$$

The PSD and the root mean square (RMS) of von Mises stress in the software Abaqus 2017 are calculated based on the reference [25]. In the software Abaqus 2017, the PSD for von Mises stress at node a is given as follows:

(4) $$S_{mises}^{a}\left(f\right)=\sum_{\beta =1}^{m}\sum_{\alpha =1}^{m}S_{\alpha \beta }\left(f\right)T_{\alpha \beta }^{a}$$

where *m* is the number of modes and $S_{\alpha \beta }(f)$ are the elements of the PSD matrix of generalized displacements.

(5) $$T_{\alpha \beta }^{a}={\left[\psi_{\alpha }^{a}\right]}^{T}\left[A\right]\left[\psi_{\beta }^{a}\right]$$

$\left[\psi_{\alpha }^{a}\right]$ are the modal stress components of the *α*th mode of node a, and the constant matrix A is given as follows:

(6) $$\left(\begin{array}{cccccc} 1 & -1/2 & -1/2 & 0 & 0 & 0\\ -1/2 & 1 & -1/2 & 0 & 0 & 0\\ -1/2 & -1/2 & 1 & 0 & 0 & 0\\ 0 & 0 & 0 & 3 & 0 & 0\\ 0 & 0 & 0 & 0 & 3 & 0\\ 0 & 0 & 0 & 0 & 0 & 3 \end{array}\right)$$

Similarly, the root mean square of the von Mises (RMS) stress of node a is computed as

(7) $$RMISES^{a}\left(f\right)=\sqrt{\sum_{\beta =1}^{m}\sum_{\alpha =1}^{m}V_{\alpha \beta }\left(f\right)T_{\alpha \beta }^{a}}$$

where $V_{\alpha \beta }\left(f\right)$ are the elements of the variance matrix of generalized displacements.

### 2.2. Estimation of Fatigue Life Under Random Vibration

Under random vibration conditions, the alternating stress or deformation will cause the fatigue cumulative damage of CHSV [26]. Linear fatigue cumulative damage assumes that the sequence of alternating load and the cumulative damage do not affect each other, which is widely used to predict the fatigue life of structures made of CFRP [27] and aluminum alloy [28]. In addition, the different types of load damage superposition are linear distributions, while fatigue damage occurs when the value of cumulative damage reaches 1. Miner’s linear cumulative damage theory is given as follows [29]:

(8) $$D=\sum D_{i}=\sum \frac{n_{i}}{N_{i}}$$

where $n_{i}$ and $N_{i}$ denote the actual number of stress cycles and the fatigue life at the stress level $S_{i}$, respectively.

In the case that the cyclic stress is continuously varying, Equation (8) can be transformed into

(9) $$D=\int_{0}^{+\infty }\frac{n_{s}}{N_{s}}ds$$

where $n_{s}$ and $N_{s}$ are the number of actual cycles and damage cycles when the peak stress is *s*, respectively. Generally when *D* = 1, the CHSV will be damaged.

The power function model is widely used to describe the fatigue life curve of common composite materials and is given as follows:

(10) $$\begin{array}{ccc} s^{m}N & = & c \end{array}$$

*c* and *m* are the material constants, obtained through fatigue testing. *N* is the number of damage cycles when the stress amplitude is *s*.

## 3. Simulation and Experimental Procedure

### 3.1. Composite Hydrogen Storage Vessel

**Figure 1:**
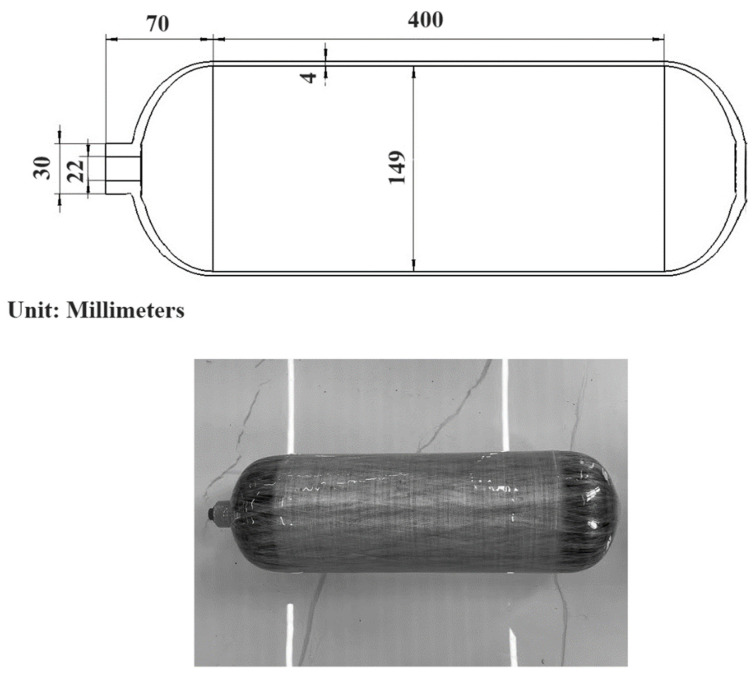
Geometric structure diagram of CHSV.

Figure 1 shows the CHSV with an inner radius of 74.5 mm and an outer radius of 78.5 mm, which is supported by a Chinese company (Shanghai, China). The CHSV consists of a thin inner liner (*h*_Al_ = 4 mm) made of aluminum alloy and an outer winding layer (*h*_CFRP_ = 3.2 mm) made of carbon fiber-reinforced composite (CFRP), which is manufactured through the dry winding process [30]. The stacking sequence of the CFRP winding layer is [13.5°_2_/90°_2_]_7_, which consists of long and continuous T700 carbon fibers and FXR-521L/FXC-521L epoxy resin. The viscosity of epoxy resin (supplied by the Shanghai Fuchen Chemical Co., Ltd., Shanghai, China), used for CHSV, is 4000 to 8000 cP at 25 °C, and the solid particle size is less than 10~25 μm. The fiber volume fraction of the CFRP winding layer is approximately 0.62, and the fiber volume fraction of the winding layer is always around 60% to 70% [31]. Material properties of the CHSV are listed in Table 1, which are provided by the Chinese company.

### 3.2. Modal Testing

Modal testing is widely used to investigate the vibrational frequency and mode of a mechanical structure, which is of great significance and the crucial step in studying the dynamic properties of CHSV under random vibration.

**Figure 2:**
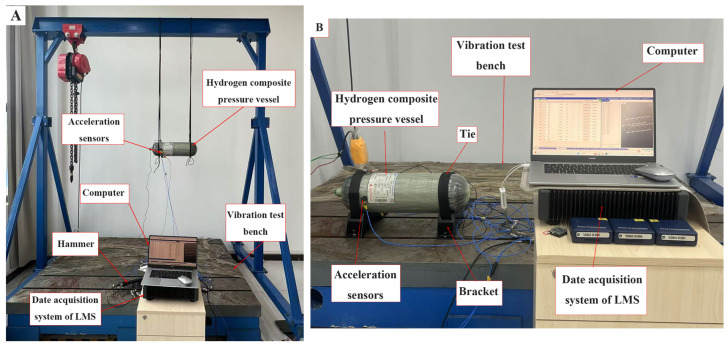
Schematic diagram of (**A**) the free modal experiment and (**B**) the constrained modal experiment of CHSV.

In our study, both free modal testing and constrained modal testing were conducted to investigate the vibration modes and frequencies of three same-type CHSVs and verify the accuracy of numerical simulation. As shown in Figure 2A, the CHSV was suspended using four elastic ropes during the free modal test. The equipment for the constrained modal test of CHSV is presented in Figure 2B. The two ends of CHSV were fixed to the brackets by the flexible tapes, and the brackets were attached to the vibration test bench through a bolted connection. Both the free and constrained modal tests were performed using the impact test method, while four PCB three-phase accelerometers were used to collect the acceleration signals of four points evenly distributed around the circumference of CHSV at every hammer blow. In order to accurately obtain the vibration characteristics of CHSV, the acceleration signals of 20 points on five equidistant sections along the CHSV axis were obtained through five impact tests sequentially. The Leuven measurement and system (LMS) data acquisition system was used to obtain and analyze the vibration patterns of CHSV at different intrinsic frequencies.

### 3.3. Finite Element Modeling

**Figure 3:**
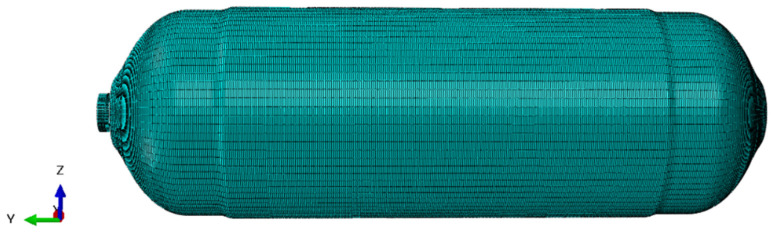
Finite element model of CHSV.

Finite element modeling is extensively used in modal analysis and random vibration analysis [32], which can simulate various test schemes and save design time. In the present study, the basically finite element model of CHSV meshed with linear hexahedral elements (C3D8R) was developed in the software Abaqus 2017, which is shown in Figure 3. The linear hexahedral elements (C3D8R) were widely used to simulate the fatigue life of engineering parts under alternating loads, which has the advantages of shortening the computation time, improving the computation accuracy, and preventing the shear self-locking phenomenon [33]. The element number of the inner aluminum alloy liner and CFRP winding layer of CHSV is 72,360 and 720,560, respectively. The geometric dimension of CHSV has been described in Section 3.1, as well as the material properties of the inner aluminum alloy liner and CFRP winding layer, are listed in Table 1.

**Figure 4:**
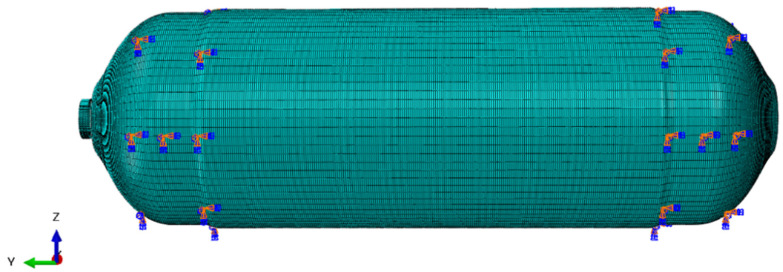
Finite element model and boundary condition of constrained mode and random vibration simulation.

For the free modal simulation of CHSV, the degrees of freedom for all nodes were set to free, while the degrees of freedom of nodes located at the fixation area of the vessel according to the constrained modal test were constrained for constrained modal simulation. The finite element model and boundary condition of CHSV under the constrained modal test and random vibration condition are presented in Figure 4.

**Figure 5:**
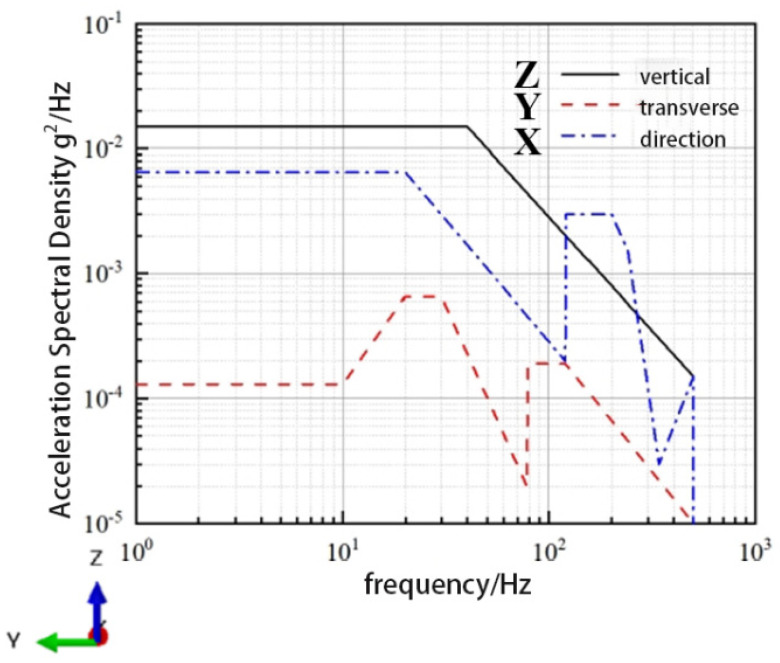
Acceleration power spectral density [34].

The random vibration simulation of CHSV was then carried out on the basis of constrained modal simulation, and a random vibration load was applied to CHSV. Furthermore, the applied random vibration load generated by a vehicle driving in a real situation was according to the reference [34] and the acceleration power spectral density of the loaded vehicle under a vibration environment is shown in Figure 5. As shown in Figure 5, the vibration in the vertical direction is the most intense, which is more representative and is consistent with the *z* direction in Figure 4.

## 4. Results and Discussion

### 4.1. Modal Simulation and Experiment

**Figure 6:**
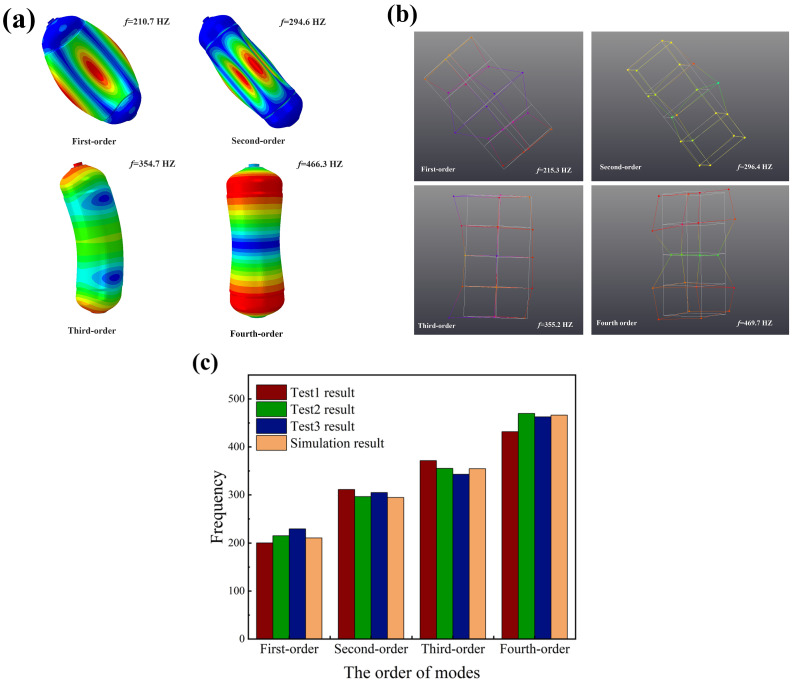
The first four orders of free modes of CHSV: (**a**) the finite element prediction of the vibration modes, (**b**) the experimental result of the vibration modes, and (**c**) the comparison of the vibration frequencies.

Figure 6 compares the finite element predictions and experimental results of the first four orders of free modes of CHSV. There is a good match of vibration pattern and frequency between the results from the finite element analysis (FEA) and from the experiment test. From Figure 6c, it can be observed that the vibration frequency of FEA and the experiment test increases from about 210 Hz to 470 Hz, and the maximum difference in the value of vibration frequency between the results from the FEA and experiment test is about 8.9% at the first-order mode. As shown in Figure 6a,b, the first four vibration patterns of CHSV are, respectively, the compression and bulge that appeared in the middle section with a symmetrical distribution of 90 degrees, the compression and bulge that presented in the middle section with a symmetrical distribution of 45 degrees, the bending in the middle section, and the compression in the middle section.

**Figure 7:**
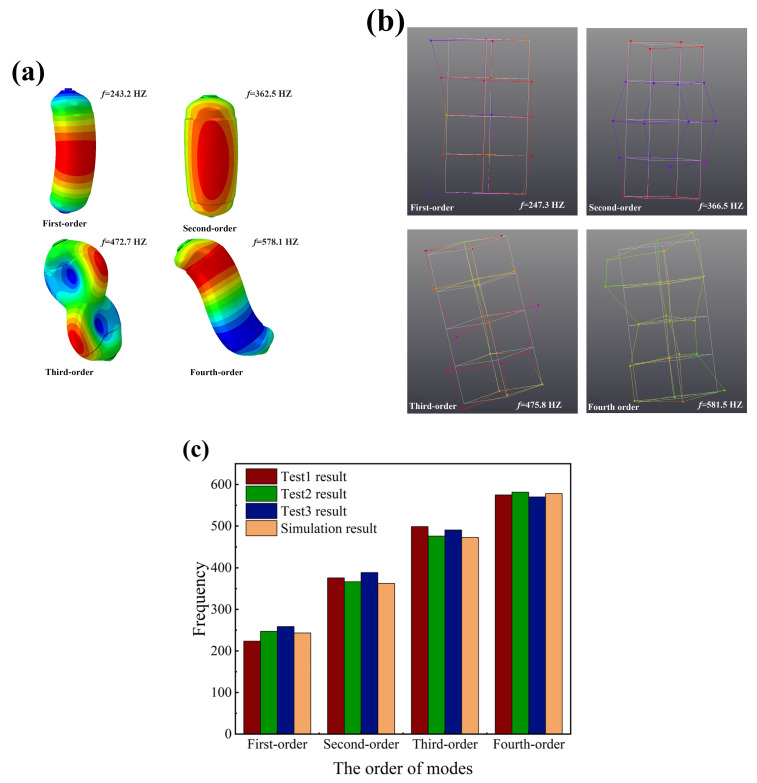
The first four-orders of constrained modes of CHSV: (**a**) the finite element prediction of vibration modes, (**b**) the experimental result of vibration modes, and (**c**) the comparison of vibration frequencies.

Figure 7 shows the comparison between the finite element predictions and experimental results of the first four orders of constrained modes of CHSV. The results of vibration pattern and frequency from FEA show a good agreement with the experimental results. From Figure 7c, it can be found that the vibration frequency of FEA and experiment test increases from about 240 Hz to 580 Hz, and the maximum difference in the value of vibration frequency between the results from the FEA and experiment test is about 8.0% at the first-order mode. Furthermore, the first-order vibration pattern is the cylinder bending in the middle section, the second-order vibration pattern is the expansion of the middle of the cylinder, the third-order vibration pattern is the depression of the upper and lower parts of the cylinder in the opposite direction, and the fourth-order vibration pattern is the twisting of the cylinder in the form of an S shape.

By comparing Figure 6 with Figure 7, it can be found that the same order vibration frequency of the constrained mode is higher than that of the free mode, while their vibration modes of each order show obvious differences. In addition, it can be concluded that the finite element model of CHSV has a high accuracy based on the comparison between the finite element predictions and experimental results of the free mode and constrained mode.

### 4.2. Random Vibration

**Figure 8:**
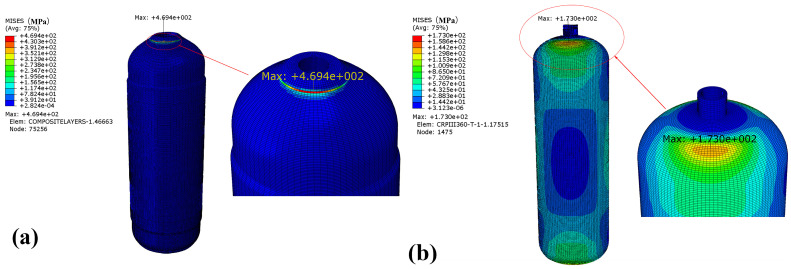
Stress of the (**a**) winding layer and (**b**) inner liner under random vibration.

The von Mises stress of the winding layer and the inner liner of the CHSV under random vibration is shown in Figure 8. It can be seen that the maximum stress points for the winding layer and inner liner are both concentrated near the CHSV mouth, where the location with the thickest fiber buildup is. The maximum value of von Mises stress is 469.4 MPa for the winding layer and 173.0 MPa for the liner, respectively. It is obvious that the winding layer is more prone to fatigue failure compared with the inner liner under random vibration.

**Figure 9:**
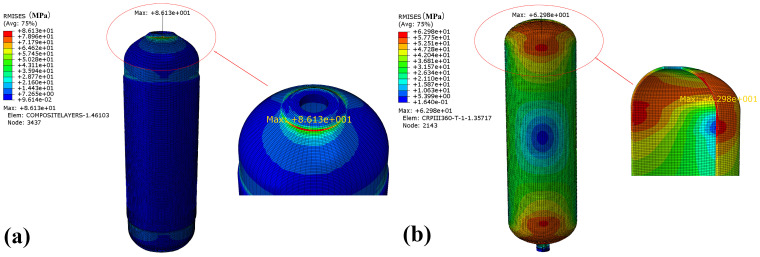
Rmises stress of the (**a**) winding layer and (**b**) inner liner under random vibration.

The root mean square of von Mises (Rmises) stress of the winding layer and the inner liner of the CHSV under random vibration is presented in Figure 9. Rmises stress is the probability distribution of von Mises stress, which is an important research reference in the random vibration study and reflects the magnitude of stress fluctuation. The maximum value of Rmises stress is 86.13 MPa for the winding layer and 62.98 MPa for the liner, respectively, which will be used for subsequent fatigue life prediction.

**Figure 10:**
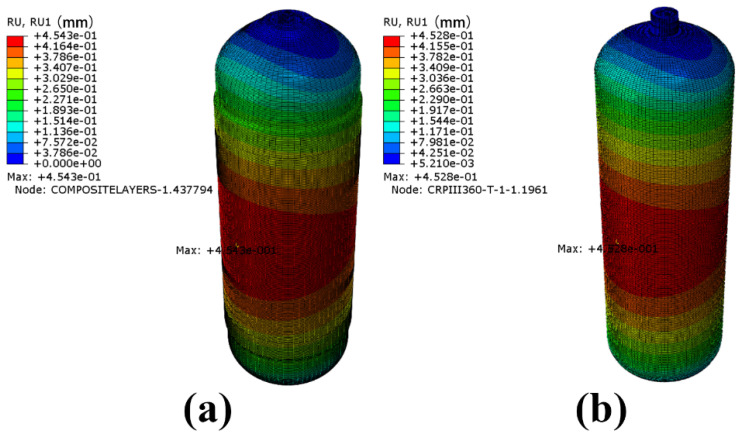
Maximum vibration deformation of (**a**) the winding layer and (**b**) the inner liner of the CHSV under random vibration.

Figure 10 shows the maximum vibration deformation of the winding layer and the inner liner of the CHSV under random vibration. The maximum values of deformation of both the winding layer and the inner liner located in the center of the vessel are about 0.45 mm and will not produce a large distortion.

### 4.3. Fatigue Life Calculations

In the present study, the Steinberg’s method is used to predict the fatigue life of CHSV. The Steinberg’s method, also known as the three-band method, assumes that the stresses follow a Gaussian distribution and the cumulative damage is a linear combination of damages under different stress levels. The cumulative damage *D* and the fatigue life *n* are defined as follows [35]:

(11) $$D=\frac{n_{1\sigma }}{N_{1\sigma }}+\frac{n_{2\sigma }}{N_{2\sigma }}+\frac{n_{3\sigma }}{N_{3\sigma }}$$

(12) $$n=\frac{1}{\frac{0.683}{N_{1\sigma }}+\frac{0.271}{N_{2\sigma }}+\frac{0.043}{N_{3\sigma }}}$$

where $n_{1\sigma }$, $n_{2\sigma }$ and $n_{3\sigma }$ are the actual number of cycles at −1*σ*~1*σ*, −2*σ*~2*σ*, and −3*σ*~3*σ* stress levels, respectively. $N_{1\sigma }$, $N_{2\sigma }$ and $N_{3\sigma }$ are the number of cycles of *S*-*N* curves at −1*σ*~1*σ*, −2*σ*~2*σ*, and −3*σ*~3*σ* stress levels, respectively. 0.683, 0.271, and 0.043 are the probability of stress distribution at −1*σ*~1*σ*, −2*σ*~2*σ*, and −3*σ*~3*σ* stress levels, respectively.

**Figure 11:**
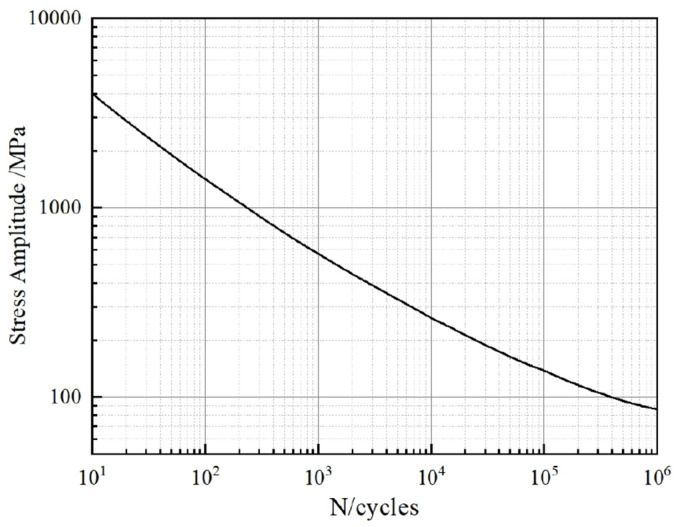
Fatigue life curve of aluminum alloy Al6061-T6 [36].

The fatigue life *S-N* curve of aluminum alloy Al6061-T6 is presented in Figure 11 [36], which is used to predict the life cycle of the CHSV inner liner made of Al6061-T6. In Section 4.2, the maximum values of stresses in the inner liner of CHSV with confidence interval 1σ, 2σ and 3σ are 62.98 MPa, 125.96 MPa, and 188.94 MPa, respectively, corresponding to the numbers of cycles $N_{1\sigma }$ = 1.04 × 10^7^, $N_{2\sigma }$ = 2.31 × 10^6^, and $N_{3\sigma }$ = 6.38 × 10^5^, respectively. According to Equation (12), the number of life cycles of the inner liner can be obtained, as follows:

(13) $$n=\frac{1}{\frac{0.683}{N_{1\sigma }}+\frac{0.271}{N_{2\sigma }}+\frac{0.043}{N_{3\sigma }}}=\frac{1}{\frac{0.683}{1.04\times 10^{7}}+\frac{0.271}{2.31\times 10^{6}}+\frac{0.043}{5.38\times 10^{5}}}=3.79\times 10^{6}$$

**Figure 12:**
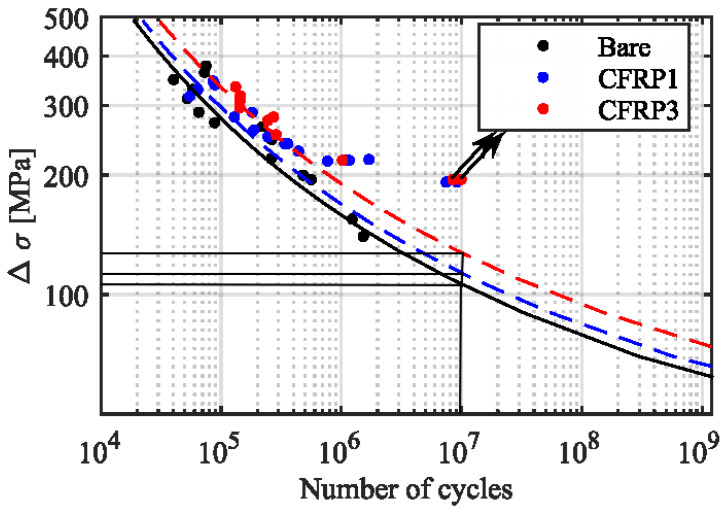
Fatigue life curve of CFRP [37].

The fatigue life *S*-*N* curve of CFRP in the winding layer is shown in Figure 12 [37], and the red line shows the fatigue life curve of the specimen reinforced with multiple layers of CFRP. In Section 4.2, the maximum values of stresses of composite layup of the CHSV with confidence interval 1σ, 2σ and 3σ are 86.13 MPa, 172.26 MPa, and 258.39 MPa, respectively, and the corresponding numbers of life cycles are $N_{1\sigma }$ = 2.14 × 10^7^, $N_{2\sigma }$ = 1.22 × 10^6^, and $N_{3\sigma }$ = 2.63 × 10^5^, respectively. The fatigue life can also be calculated according to Equation (12), as follows:

(14) $$n=\frac{1}{\frac{0.683}{N_{1\sigma }}+\frac{0.271}{N_{2\sigma }}+\frac{0.043}{N_{3\sigma }}}=\frac{1}{\frac{0.683}{2.14\times 10^{7}}+\frac{0.271}{8.97\times 10^{5}}+\frac{0.043}{1.92\times 10^{5}}}=1.79\times 10^{6}$$

**Figure 13:**
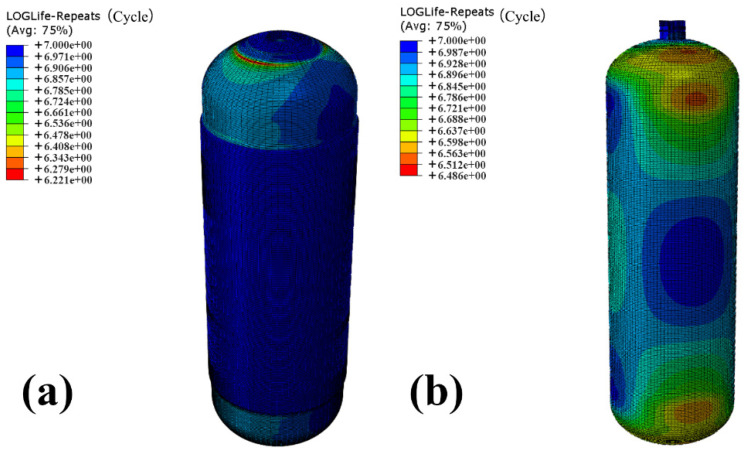
Fatigue life of (**a**) the winding layer and (**b**) the inner liner of the CHSV using software Fe-safe.

In addition, the Rmises stress results of the winding layer and the inner liner obtained in Section 4.2 were then imported into software Fe-safe 2017, in which Dirlik’s method was adopted to calculate their fatigue life. Figure 13 shows the results of the fatigue life of the winding layer and the inner liner calculated from the software Fe-safe. The numbers of cycles of the winding layer and the inner liner calculated are 10^6.221^ = 1.66 × 10^6^ and 10^6.486^ = 3.06 × 10^6^, respectively. Furthermore, the lowest fatigue life area of the winding layer appears at the CHSV neck, as well as the lowest fatigue life area of the inner liner located at the two ends of CHSV.

The fatigue life of the winding layer and the inner liner calculated by the Steinberg’s method and the Dirlik’s method are compared in Table 2. The values of the number of life cycles of the winding layer and the inner liner calculated by the Steinberg’s method are slightly higher than the results calculated by the Dirlik’s method, and the two methods obtain similar prediction results, indicating the accuracy of FEA of fatigue life in the software Fe-safe. Both the values of the number of life cycles of the winding layer and the inner liner are lower than 1 × 10^7^ (high cycle fatigue life standard). In order to further improve the fatigue life of CHSV, the structural optimization of CHSV was then carried out in Section 4.4.

### 4.4. Structural Optimization and Analysis

The most effective and common way to optimize the structure of CHSV is to adjust the spiral winding angle and reduce the thickness of the head section [38]. Reaming is a process to improve the fiber thickness distribution of the head section by changing the helical winding angle, which can make the strength of the head section be distributed more uniformly and improve the bearing capacity of CHSV, without considering the hydrogen embrittlement. Due to the limitation of the curved surface of the head section, the helical winding angle generally ranged from 12° to 70° [39]. Furthermore, the number of layers must be even and consistent based on the characteristics of spiral winding. The above constraint conditions are given as follows [40]:

(15) $$\{\begin{array}{c} 13.5^{\circ }\leq \alpha \leq 70^{\circ }\\ \alpha_{i}<\alpha_{i+1}\\ \frac{b}{2R}<\sin\alpha_{i+1}-\sin\alpha_{i}<\frac{2b}{R}\\ \sum n_{i}=x \end{array}$$

where *i* = l, 2, … 6, *x* is the number of spiral winding layers, and α the helical winding angle.

In the reaming process, the determining factor of the final thickness distribution of the winding layer is the reaming angle and the corresponding number of winding layers. In the present study, as listed in Table 3, six kinds of winding angles are chosen.

**Figure 14:**
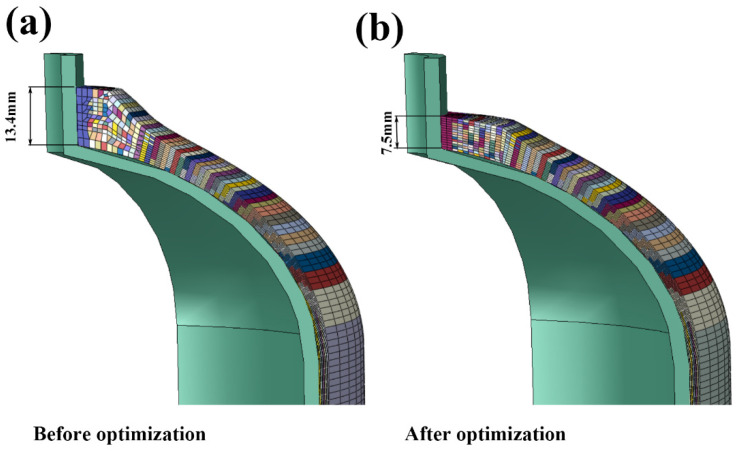
Comparison of winding layers: (**a**) before optimization and (**b**) after optimization.

The comparison of winding layers before and after optimization is shown in Figure 14. It can be found that the angle of the winding layer and the stacking of fibers become smoother after the redesign and optimization. The thickness of the fiber layer of the head section is about 13.4 mm before optimization, which is reduced to 7.5 mm after optimization with an obvious decrease of 44%.

**Figure 15:**
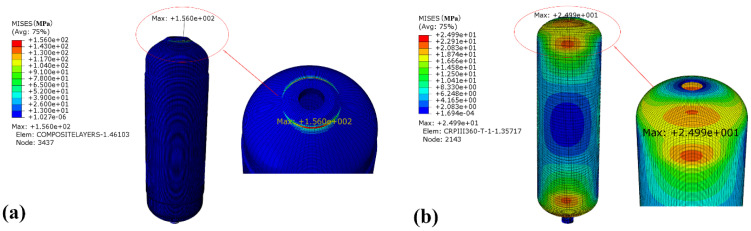
Stress of (**a**) the winding layer and (**b**) the inner liner of the CHSV after optimization.

Figure 15 shows the stress of the winding layer and the inner liner of the CHSV after optimization under the same vibration conditions. Compared with Figure 8, it can be observed that the stresses are still concentrated on the head section of CHSV. However, the maximum value of stress of the winding layer decreases from 469.4 MPa to 156.0 MPa, as well as the maximum value of stress of the inner liner, which decreases from 173.0 MPa to 25.0 MPa. The reduction amplitudes of the maximum stresses of the winding layer and the inner liner reached 66% and 85%, respectively, indicating that the optimization of the winding layer can integrally improve the bearing capacity of CHSV. Similar results of stress reduction after the optimization of the winding layer have been published in the references [41,42].

**Figure 16:**
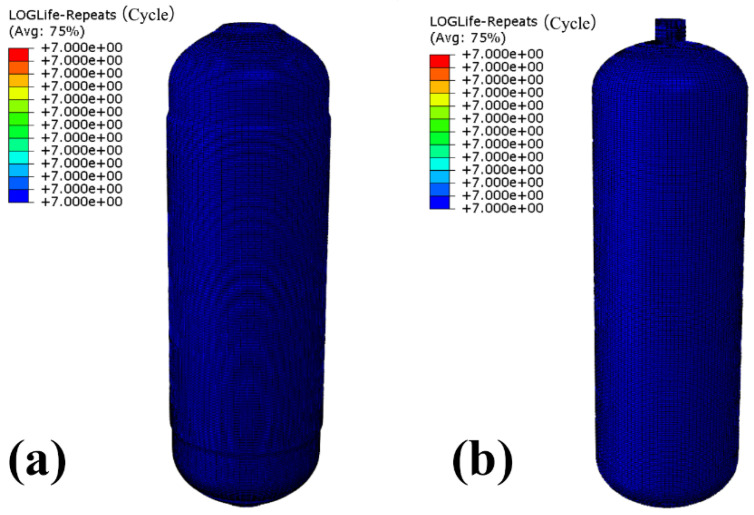
The fatigue life of (**a**) the winding layer and (**b**) the inner liner of the CHSV after optimization.

Fatigue life prediction of optimized CHSV is presented in Figure 16. The number of life cycles of the winding layer and of the inner liner have both been greatly improved, reaching over 1 × 10^7^ (high cycle fatigue life standard). It can be concluded that the optimization of the lay-up angle can effectively improve the safety of the CHSV under random vibration.

## 5. Conclusions

In this paper, the finite element models of CHSV are developed to simulate their dynamic response under the modal testing condition and random vibration condition. Modal testing experiments are conducted to verify the accuracy of numerical simulations. Based on the simulation results of stress under random vibration, the fatigue life of CHSV is subsequently calculated by Steinberg’s method and Dirlik’s method, respectively. In the end, the optimization of ply parameters of the composite winding layer was conducted to improve the stress distribution and fatigue life of CHSV. Based on the studies, the following conclusions are drawn:

1. The predicted results of the vibration pattern and frequency of CHSV under the free mode and constrained mode conditions show a good agreement with that of the experiment tests. The maximum difference in the value of the vibration frequency for the free and constrained modes of CHSV from the FEA and experiment tests both occur at the first mode and are 8.9% and 8.0%, respectively, verifying the accuracy of the finite element modeling of CHSV;
2. Without the optimization of the winding layer, the maximum values of stress are 469.4 MPa for the winding layer and 173.0 MPa for the liner under random vibration, and both appear at the head section with the thickest fiber accumulation, which indicates that the head section is most likely to occur fatigue failure. Through the optimization of the winding layer, the thickness of the fiber layer of the head section is reduced from 13.4 mm to 7.5 mm, with an obvious decrease of 44%. In addition, the stress of the winding layer and the inner liner after the optimization of the winding layer dropped by 66% and 85%, respectively;
3. There is no obvious difference between the fatigue life of the winding layer and the inner liner calculated by Steinberg’s method and Dirlik’s method. Without the optimization of the winding layer, the number of life cycles of the winding layer and the inner liner under random vibration conditions obtained from Dirlik’s method is about 1.66 × 10^6^ and 3.06 × 10^6^, respectively, while the lowest fatigue life area of the winding layer appears at the CHSV neck and the lowest fatigue life area of the inner liner located in the two ends of CHSV. After the optimization of the winding layer, the numbers of life cycles of the winding layer and the inner liner have been significantly improved, and both reached over 1 × 10^7^ (high cycle fatigue life standard).

The research in this paper provides theoretical guidance for the design, modeling, and optimization of CHSV under random vibration of CHSV, which can be extrapolated to other vessels consisting of the CFRP winding layer and inner liner made of metal materials or polymer materials.

## Figures and Tables

**Table 1:** Material properties of the CHSV.

Material Properties	CFRP (T700/Epoxy)	Aluminum Alloy (6061-T6)
Longitudinal modulus, *E*_1_ (GPa)	139.00	6.00
Transverse modulus, *E*_2_ = *E*_3_ (GPa)	8.00	
Longitudinal shear modulus, *G*_12_ = *G*_13_ (GPa)	6.37	
Transverse shear modulus, *G*_23_ (GPa)	3.70	
Major Poisson’s ratio, *υ_f_*_12_ = *υ_f_*_13_	0.30	0.30
Minor Poisson’s ratio, *υ_f_*_23_	0.35	

**Table 2:** Random vibration fatigue life estimation results.

Fatigue Life Estimation	Winding Layer	Inner Liner
Number of life cycles (Steinberg method)	1.79 × 10^6^	3.79 × 10^6^
Number of life cycles (Dirlik method)	1.66 × 10^6^	3.06 × 10^6^

**Table 3:** Optimized layer laying scheme.

Winding Angle	13.5°	24.1°	36.6°	47.8°	55.5°	90.0°
Number of winding layers	6	2	2	2	2	16

## Data Availability

The original contributions presented in this study are included in the article. Further inquiries can be directed to the corresponding author.
